# A cross-sectional study of dietary and urinary soy isoflavones about coal-burning fluorosis in Guizhou, China

**DOI:** 10.3389/fnut.2025.1589177

**Published:** 2025-07-24

**Authors:** Hua Han, Yameng Li, Peimin Li, Lu Liu, Xingting Zheng, Yuanmei Zhang, Min Liu, Xiaogang Zhang, Ling Tang, Manman Gao, Na Tao, Jun Liu

**Affiliations:** ^1^Department of Clinical Nutrition, The First People’s Hospital of Zunyi (The Third Affiliated Hospital of Zunyi Medical University), Zunyi, China; ^2^Department of Preventive Medicine, School of Public Health, Zunyi Medical University, Zunyi, China; ^3^Department of Chronic Disease, Center of Disease Control and Prevention of Honghuagang District, Zunyi, China; ^4^Department of Pharmacy, Affiliated Hospital of Zunyi Medical University, Zunyi, China; ^5^Key Laboratory of Maternal & Child Health and Exposure Science of Guizhou Higher Education Institutes, Zunyi, China

**Keywords:** soy isoflavones, dietary, coal-burning fluorosis, cross-sectional study, factor analysis

## Abstract

**Objective:**

Previous research showed soy isoflavones have antioxidant properties beneficial to bone health, but no epidemiological studies reported effects of soy isoflavones on fluorosis. Therefore, the aim of this study was to investigate the relationship between soy isoflavones in diet/urine and coal-burning fluorosis.

**Methods:**

A comprehensive cross-sectional study with 896 participants in Zhijin County, Guizhou, China, assessed dietary intake through face-to-face interviews using a 75-item food frequency questionnaire. Urine samples were analyzed for soy isoflavone concentrations by HPLC. Unconditional logistic regression models were used to calculate odds ratios (ORs) and 95% confidence intervals (CIs) for associations.

**Results:**

We observed a significant inverse association between dietary soy isoflavones and fluorosis. The adjusted OR (95% CI) in the highest quartile of intake compared with the lowest was 0.61 (0.38–0.97) (*p*-trend = 0.032) for total soy isoflavones intake, 0.59 (0.37–0.95) (*p*-trend = 0.032) for daidzein intake. Results of soy isoflavones in urine were consistent with the dietary results.

**Conclusion:**

Soy isoflavones are associated with the occurrence of coal-burning fluorosis.

## 1 Introduction

Endemic fluorosis is a condition caused by exposure to high concentrations of fluoride in the environment, primarily manifested as dental fluorosis and skeletal fluorosis. Furthermore, in recent years, theories regarding the pathogenesis of systemic damage induced by endemic fluorosis have emerged (1). This condition has a prevalent range that spans across 13 provinces, with a concentration in areas such as Yunnan, Guizhou, and Sichuan, affecting over 34.3 million people and posing as a worldwide public health issue (2, 3). Although the cases of fluorosis from coal-burning have been reduced through the using of improved stoves and awareness education, the prevalence of fluorosis is still high (4–7). Therefore, further prevention and control of fluorosis is needed.

Soy isoflavones are derived from soy foods, a secondary metabolite in the process of soybean growth, which has antioxidant biological activity and can alleviate cellular oxidative stress (8). Studies have shown that soy isoflavones have a protective effect against oxidative stress-related diseases due to their antioxidant activity, in addition to being closely associated with bone health (9, 10). A large number of observational studies have shown that the higher the intake of soy isoflavones, the higher the bone mineral density (BMD) or the lower the bone resorption markers (11). The latest meta-analyses showed that soy isoflavones exert beneficial effects on bone formation markers that can prevent osteoporosis-related bone loss in any weight status or treatment duration (12–14). Isoflavones may benefit bone because they have positive effects on the osteoblast cell (favoring proliferation, differentiation and mineralization) and hinder osteoclast and adipocyte generation (15, 16). Fluorosis is a disease affecting mainly skeletal system and oxidative stress has been considered as an important pathogenesis of endemic fluorosis (17). Based on the positive effects of soy isoflavones on bone health and antioxidant activity, it is suggested that soy isoflavones may have a certain beneficial effect on fluorosis. Furthermore, animal experiments have found that soy isoflavones can inhibit the lipid peroxidation of liver tissue, enhance the antioxidant capacity, and have protective effects on oxidative damage in fluorosis rats (18), but no epidemiological study has confirmed it.

Therefore, we conducted a cross-sectional study to investigate the associations of external (dietary intake) and internal (urine) exposure levels with fluorosis in coal-burning fluorosis areas of Guizhou, China.

## 2 Materials and methods

### 2.1 Study subjects

A population-based cross-sectional study was conducted in a coal-burning area of Zhijin County, Guizhou Province, China. We used a two-stage, clustered random sampling method to ensure the representativeness of the target population. The three towns of Chadian, Chengguan, and Puweng were randomly selected from 10 towns in Zhijin County. Then, we randomly further selected four villages from each selected town. The 12 villages selected for the study were Dazai, Ganhe, Gaofeng, Guihua, Guohua, Hehua, Hualuo, Jiangyan, Moda, Shangzai, Yutang, and Xianfeng. Participants who have lived in Zhijin County for at least 10 years and aged 18–75 years were recruited. Participants were excluded if they had a prior history of cancer, coronary heart disease, stroke, gout, or kidney disease. They were also excluded if their dietary habits had manifestly changed during the previous 5 years, or if they had chronic diseases that might affect their dietary habits, such as gastritis, diabetes, and hypertension. In addition, the participants with incomplete questionnaire information were also excluded. Finally, a total of 896 participants were successfully interviewed out of 1,101 recruited. The urine samples of 720 subjects were obtained from the 896 participants interviewed. It was reported the prevalence of coal-burning fluorosis in Guizhou was 35% (19), allowable error *d* = 0.15*p*, and significance level *α* = 0.05; $n=\frac{Z_{\alpha /2}\times p\times (1-p)}{d^{2}}$ . The sample size was estimated to be 318 cases based on the sample size estimation formula for cross-sectional studies.

All of the coal-burning fluorosis cases were diagnosed by the Zhijin County Disease Control and Prevention Center according to the Chinese Diagnostic Criteria of Dental Fluorosis (WS/T208-2011, China) and the Chinese Diagnostic Criteria of Endemic Skeletal Fluorosis (WS/T 192-2008, China) (20). Specifically, dental fluorosis was confirmed in individuals with a documented history of excessive fluoride exposure during tooth development, presenting with at least one enamel defect: white non-removable opacities, intrinsic brown/black-brown discoloration, or pitted/map-like hypoplasia. Similarly, skeletal fluorosis was diagnosed in individuals with a documented history of residence in an endemic fluorosis area, presenting with persistent resting pain in ≥3 bony/joint sites (unaffected by weather), joint limitation/neurological deficits (limb deformities, paralysis). Dental fluorosis and skeletal fluorosis were identified by a professional doctor in Zhijin Center for Disease Control and Prevention. Concurrently, non-fluorosis was defined as the absence of pathological changes in individuals, such as dental fluorosis or skeletal fluorosis, despite long-term living in fluorosis area.

This study complied with the Declaration of Helsinki guidelines and was approved by the Zunyi Medical University Medical Ethics Committee (No. 2014-1-003), with all participants providing written informed consent. Using cluster sampling of villages in coal-burning fluorosis-endemic areas, we ensured comprehensive representation of the target population. This approach, combined with a broad age range, redundant sample size, and standardized diagnostics, effectively mitigated selection bias.

### 2.2 Data collection pertaining to diet and lifestyle

Interviews were conducted by trained interviewers who administered a structured questionnaire in a personal interview. The content of the questionnaire included: (1) socio-demographic characteristics (age, gender, ethnicity, marital status, and education level); (2) lifestyle habits (use of improved stoves, domestic fuel type, use of coal to roast grains and chilies, and washing dry grains and chilies before use); (3) dietary habits in the year before the interview; (4) relevant disease history (hypertension, diabetes, gout, hepatocirrhosis, heart-related diseases). Dietary intake of nutrients intake was collected through a valid and reliable Food-Frequency Questionnaire (FFQ) (21) and assessed never, daily, weekly, monthly, or yearly frequency intake of each food item over the last year. The 75-items FFQ involved seven categories including fruits, animal food, cereals, beans and their products, vegetables, algae and nuts, drinks and soup. The FFQ covered virtually all soy foods that are habitual consumed in the study population, including firm tofu, soft tofu, bean curd skin, soy drink, tofu pudding and fresh and dried soybeans. Energy, soy isoflavones, genistein, daidzein and glycitein and other nutrient intakes were calculated using the Chinese Food Composition Database (22).

### 2.3 Detection of urinary levels of soy isoflavones

Level of glycitein, genistein, daidzein, dihydrodaidzein, equol, and O-desmethylangolensin in the urine were detected using a high-performance liquid chromatography (HPLC) methods. Briefly, Urine samples were extracted by ethyl acetate after deconjugation by β-glucuronidase/sodium acetate. After nitrogen drying, the extract was reconstituted in mobile phase solution for analysis. The HPLC system consisted of a C18 stationary phase extraction (5 μm, 4.60 mm × 250 mm) column, and separation of soy isoflavones was achieved by gradient elution with the mobile phase of methanol-ethyl acetate at a flow rate of 1.0 mL/min. Soy isoflavones were detected from the UV absorbance at 254 nm. The standards of glycitein, genistein, daidzein, dihydrodaidzein, equol, O-desmethylangolensin and β-glucuronidase were purchased from Sigma Chemical Company (St. Louis, MO, United States).

### 2.4 Statistical analysis

The data were coded and doubly entered by two data clerks into Epi-Data version 3.1 to avoid clerical errors using side-by-side comparison, and the data were then exported to SPSS for windows version 18 statistical software.

Continuous variables were expressed as mean (M) and SD (s) or median (P25, P75), while categorical variables were expressed as numbers and percentages. Dietary intake of soy isoflavones showed skewed distribution, which was classified into quartiles, and the lowest quartile was used as the reference category. Logistic regression model was used to estimate the relationship between dietary intake of soy isoflavones and coal-burning fluorosis, and the odds ratio (OR) and 95% confidence interval (95% CI) were calculated. The multivariate models were used to adjust the potential confounding factors, such as age, gender, ethnicity, marital status, education level, smoking, alcohol drinking, tea drinking, improved stove use, fuel type and using coal to roast grains and chilies, washing dry grains and chilies before use (Model 1). Subsequent models were adjusted for dietary factors, such as roasted chili consumption, dietary calcium intake, total energy intake, and roasted corn intake (Model 2). Stratified analysis was performed by sex (male/female) to explore whether the association between soy isoflavones and fluorosis was modified. Subgroup analyses for dental fluorosis or skeletal fluorosis were performed for the relationships between soy isoflavones and fluorosis. We also performed sensitivity analysis to assess whether the results of primary analysis were driven by type of fluorosis. All *p*-values were two tailed, and the level of significance was set at ≤ 0.05.

## 3 Results

### 3.1 General characteristics of subjects

Of the 896 participants (414 men and 482 women), 655 were diagnosed with endemic fluorosis and 241 without fluorosis. Table 1 shows the baseline demographic characteristics of the study subjects and selected risk factors for endemic fluorosis. The mean (±) age of fluorosis group was 50.32 years old (±12.41 years old), and that of non-fluorosis group was 47.10 years old (±14.52 years old). Compared with non-fluorosis subjects, fluorosis patients tended to have lower levels of education and annual household income. Moreover, fluorosis patients were less likely to use improved cook stoves, were more likely to use coal to roast grains and chilis, and use mixed coal fuel types. Compared with non-fluorosis subjects, the intake of soy isoflavone in fluorosis patients was lower, and the level of urinary fluoride was higher.

**Table 1:** Socio-demographic characteristics and selected risk factors of the participants in coal-burning fluorosis area in Guizhou.

Characteristics	Non-fluorosis (*n* = 241)	Fluorosis (*n* = 655)	*T*/*χ*^2^/*Z*	*p* ^a^
Age, years (mean ± s)	47.10 ± 14.52	50.32 ± 12.41	3.283	0.001
Gender, *n* (%)			2.449	0.118
Female	140 (58.09)	342 (52.21)		
Male	101 (41.91)	313 (47.79)		
Marital status, *n* (%)			4.881	0.087
Married or cohabitation	199 (82.60)	554 (84.60)		
Divorce or separation	23 (9.50)	73 (11.10)		
Unmarried	19 (7.90)	28 (4.30)		
Educational years, *n* (%)			60.368	<0.001
≤3 years	84 (34.90)	339 (51.80)		
4–6 years	74 (30.70)	226 (34.50)		
7–11 years	55 (22.80)	76 (11.60)		
≥12 years	28 (11.60)	14 (2.10)		
Income (yuan/per capita/month), *n* (%)			10.804	0.013
≤500	9 (3.70)	62 (9.50)		
501–2000	96 (39.80)	265 (40.50)		
2001–4,000	64 (26.60)	178 (27.20)		
>4,000	72 (29.90)	150 (22.90)		
Smoker^b^, *n* (%)	85 (35.30)	279 (42.60)	3.920	0.048
Alcohol drinker^c^, *n* (%)	73 (30.30)	204 (31.20)	0.067	0.796
Tea drinker^d^, *n* (%)	90 (37.30)	250 (38.30)	0.066	0.797
Using an improved stove^e^, *n* (%)	209 (86.70)	465 (71.30)	22.554	<0.001
Fuel type, *n* (%)			18.301	<0.001
Raw coal	36 (56.40)	394 (60.40)		
Mixed coal	41 (17.00)	152 (23.30)		
Firewood	17 (7.10)	15 (2.30)		
Others	47 (19.50)	91 (14.0)		
Using coal to roast grains and chilis^f^, *n* (%)	114 (47.30)	390 (59.80)	11.206	0.001
Washing dry grains and chilis before use, *n* (%)	227 (96.20)	608 (93.80)	2.905	0.234
Total energy intake (kcal/day)	2538.60 (2008.76, 3210.36)	2615.86 (1992.06, 3420.85)	0.737	0.461
Calcium intake (mg/day)	481.91 (335.71, 710.39)	449.13 (314.42, 655.27)	1.790	0.074
Total protein intake (mg/day)	72.17 (55.09, 107.84)	75.47 (54.33, 100.52)	0.406	0.684
Total fat intake (mg/day)	97.65 (71.94, 147.03)	101.41 (73.01, 150.86)	0.567	0.571
Corn intake (g/day)	13.69 (4.72, 28.49)	13.69 (4.11, 32.87)	0.286	0.775
Dry chilis intake (g/day)	5.78 (2.41, 12.54)	5.78 (2.41, 14.46)	0.537	0.591
Urinary fluoride (mg/L)	1.17 (0.81, 1.65)	1.41 (1.03, 2.08)	4.703	<0.001
Soy isoflavones (mg/day)	35.39 (16.26, 72.47)	27.00 (13.82, 55.21)	2.575	0.010

a Between fluorosis and non-fluorosis differences on categorical variables were analyzed with chi-square tests, and on continuous variables were with two independent sample *t*-tests, incasing of skewed distributions, non-parametric *t*-test were used. s, SD; *n* (%), number of cases (proportion).

b Smokers were defined as smoking at least five packs of cigarettes per year.

c Alcohol drinkers were defined as having beer, white wine, or red wine at least once weekly over the past year.

d Tea drinkers were identified as having tea at the least two times per week.

e Improve open oven to reduce fluoride pollution indoors by excluding fluoride out of room.

f Burning fluoride-rich coal for roasted grain and chili.

### 3.2 Comparison of dietary and urinary isoflavones in participants with fluorosis and non-fluorosis

Compared with fluorosis patients, the dietary intake of soy isoflavones was higher in non-fluorosis subjects, and the intake of genistein was the largest in the subclass of soy isoflavones. In addition, compared with fluorosis patients, the contents of soy isoflavone and daidzein metabolites in urine of non-fluorinated subjects were higher, and equol content was the highest in urine (Table 2).

**Table 2:** Comparison of dietary intake and urinary levels of soy isoflavone between fluorosis and non-fluorosis^a^.

Soy isoflavones	Non-fluorosis group median (25th, 75th)	Fluorosis group median (25th, 75th)	*Z*	*p* ^b^
Dietary intake (mg/day)	*N* = 241	*N* = 655		
Glycitein	1.75 (0.87, 3.84)	1.35 (0.68, 2.87)	2.771	0.006
Genistein	19.41 (8.84, 39.59)	14.52 (7.54, 30.69)	2.580	0.010
Daidzein	14.46 (6.53, 29.03)	11.26 (5.57, 22.18)	2.506	0.012
Total isoflavones	35.37 (16.10, 72.22)	27.38 (13.74, 55.12)	2.571	0.010
Urinary level (ng/mL)	*N* = 192	*N* = 528		
Glycitein	22.78 (14.76, 32.13)	17.94 (12.84, 26.41)	4.194	<0.001
Genistein	22.75 (12.21, 32.33)	19.92 (10.99, 31.69)	1.574	0.116
Daidzein	26.28 (15.82, 44.06)	18.32 (12.44, 33.09)	4.956	<0.001
Dihydrodaidzein	17.36 (7.01, 39.14)	9.78 (4.31, 21.36)	5.167	<0.001
Equol	28.57 (16.69, 61.00)	21.78 (12.03, 39.12)	3.910	<0.001
O-Desmethylangolensin	1.36 (0.79, 2.79)	1.31 (0.67, 2.30)	1.230	0.219

a Soy isoflavones, genistein, daidzein and glycitein and other nutrient intakes were calculated using the Chinese Food Composition Database. Level of glycitein, genistein, daidzein, dihydrodaidzein, equol, and O-desmethylangolensin in the urine were detected using a high-performance liquid chromatography (HPLC) methods. P25 and P75 represent the 25th and 75th percentile.

b Wilcoxon rank sum test was used to compare the median consumption levels between fluorosis and non-fluorosis.

### 3.3 Associations between dietary soy isoflavones and coal-burning fluorosis

**Figure 1:**
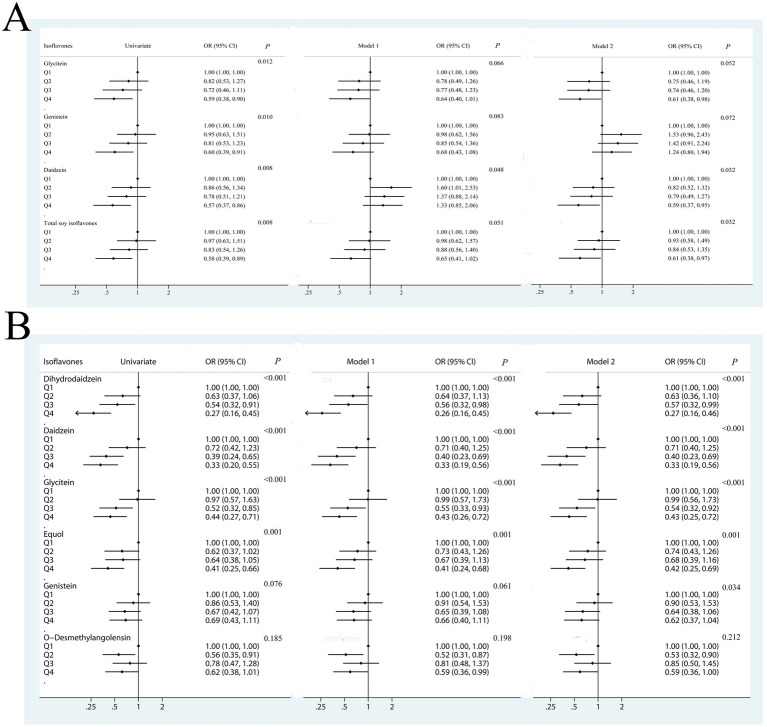
Odds ratios (ORs) and 95% CIs of fluorosis for quartiles of soy isoflavones. **(A)** ORs and 95% CIs of fluorosis for quartiles of dietary intake of soy isoflavones. **(B)** ORs and 95% CIs of fluorosis for quartiles of urinary level of soy isoflavones. OR, odds ratio; CI, confidence interval. Quartile 1 was the reference quartile. The multivariate logistic regression model was used to estimate the association between dietary intake of soy isoflavones and fluorosis. Model 1: OR was adjusted for covariates, such as age, sex, marital status, education level, income, smoking status, alcohol drinking status, tea drinking status, using coal to roast grains or chili, washing dry grains or chili before use, fuel type, and using improved stove. Model 2: OR was adjusted for the various above confounders and calcium intake, roasted chili and grains consumption, and total energy intake.

**Figure 2:**
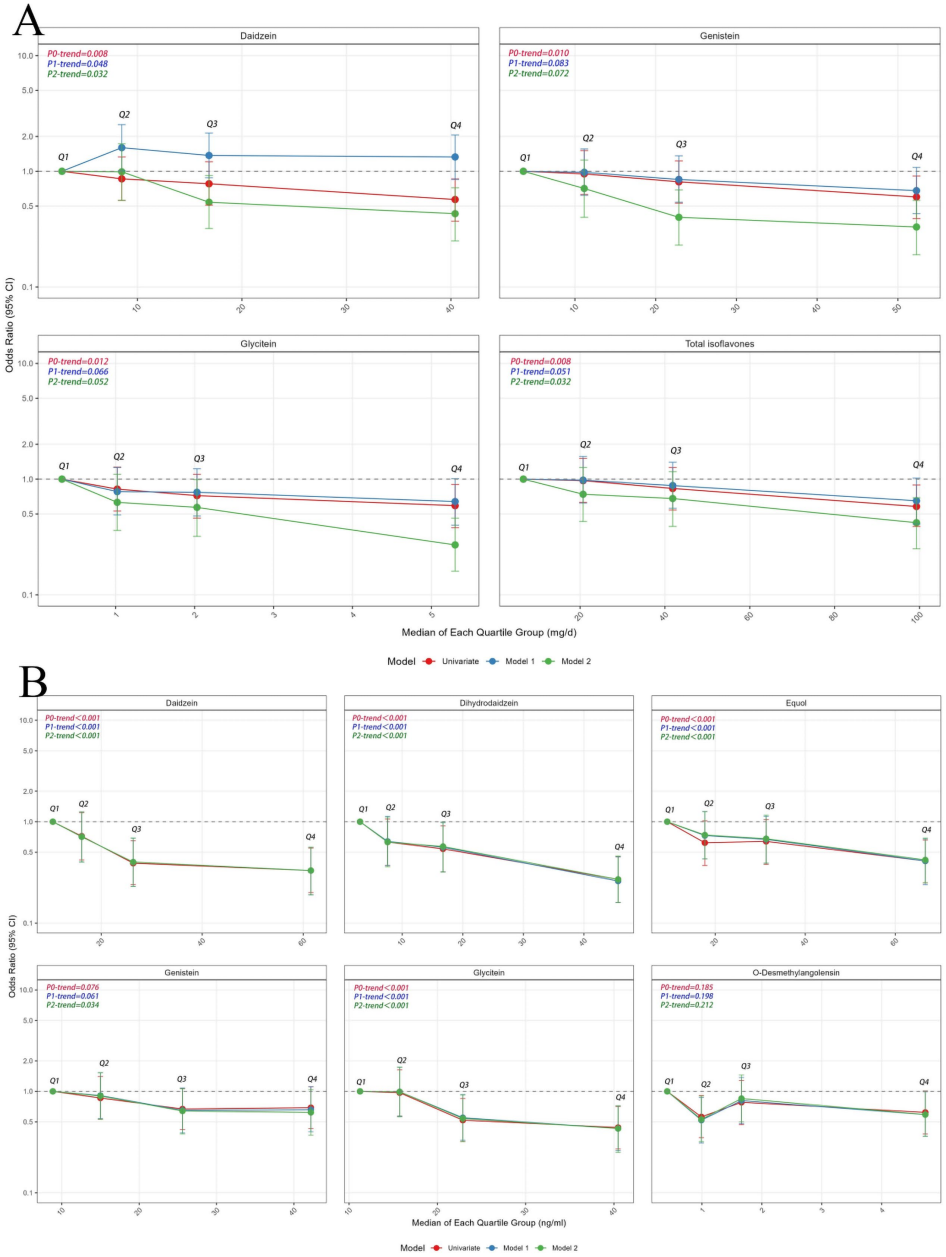
Dose–response relationship plots for soy isoflavone and occurrence of fluorosis. **(A)** Dose–response relationship plots for dietary of soy isoflavone and occurrence of fluorosis. **(B)** Dose–response relationship plots for urinary level of soy isoflavone and occurrence of fluorosis. CI, confidence interval. Q, quartile. Q1–Q4 represent four equal groups of dietary soy isoflavone intake (Q1 = lowest to Q4 = highest) and their medians. *p*-trend: test the dose–response trend between isoflavone intake and fluorosis risk. Univariate (red plot): univariate model: no covariates were adjusted. Model 1 (blue plot) adjusted for age, sex, marital status, education level, income, smoking status, alcohol drinking status, tea drinking status, using coal to roast grains or chili, washing dry grains or chili before use, fuel type, and using improved stove. Model 2 (green plot) adjusted for the various above confounders and calcium intake, roasted chili and grains consumption, and total energy intake.

Figure 1A presents the ORs and 95% CIs for fluorosis according to quartiles of dietary soy isoflavone intakes. The quartiles of dietary intake of soy isoflavones from low to high were 6.64 mg/day, 20.72 mg/day, 41.84 mg/day, 99.29 mg/day. With the increase of intake, the proportion of non-fluorosis poisoning increased. Univariate analysis showed that the OR value decreased gradually with the increase of nutrient intake, and the difference between the highest quartile and the lowest quartile of nutrient intake was statistically significant (*p* = 0.008). After further adjustment for dietary factors such as total energy, calcium intake, grains intake, and dry chilis intake, the negative associations between soy isoflavone intake and the risk of developing coal-burning fluorosis were not changed. The ORs for fluorosis occurrence in the highest quartile compared with the lowest quartile of intake 0.59 (95% CI: 0.37–0.95) for daidzein, 0.61 (95% CI: 0.38–0.97) for total soy isoflavones. Figure 2A indicates the higher dietary intake of daidzein and total isoflavones, the lower the occurrence of fluorosis (both *p*-trend = 0.032).

### 3.4 Associations between urinary soy isoflavones and coal-burning fluorosis

For the six soy isoflavone subgroups, compared to the lowest quartile of urinary isoflavone, the ORs for fluorosis occurrence in the highest quartile were 0.27 (95% CI: 0.16–0.0.46) for dihydrodaidzein, 0.33 (95% CI: 0.19–0.56) for daidzein, 0.43 (95% CI: 0.25–0.72) for glycitein and 0.42 (95% CI: 0.25–0.69) for equol (Figure 1B). Figure 2B shows the higher levels of dihydrodaidzein, daidzein, glycitein and equol in the urine, the lower the occurrence of fluorosis after adjusting for various dietary and non-dietary factors (*p*-trend <0.001–0.034). In contrast, no statistical association was observed between O-desmethylangolensin and fluorosis (*p*-trend = 0.666).

### 3.5 Sex-stratified analysis of the relationship between soy isoflavone and coal-burning fluorosis

As shown in Table 3 subgroup analysis showed there were negative associations of total soy isoflavones, glycitein, genistein and daidzein with fluorosis in women (*p* = 0.013–0.030), but not in men. We observed interactions between dietary soy isoflavone and gender on fluorosis, such as daidzein (*p*-interaction = 0.009), genistein (*p*-interaction = 0.012), glycitein (*p*-interaction = 0.011), total isoflavones (*p*-interaction = 0.012). Furthermore, there were interactions between urinary O-desmethylangolensin and fluorosis (*p*-interaction = 0.018) (Table 4 and Supplementary Figure 1). These results indicated effects of different types of soy isoflavones on fluorosis may be vary between men and women.

**Table 3:** Sex-stratified analysis of the relationship between dietary intake of soy isoflavone and fluorosis OR (95% CI).

Soy isoflavones	Quartiles				*p*-trend	*p*-interaction
	Q1	Q2	Q3	Q4		
Total isoflavones						0.012
Non-fluorosis/fluorosis	52/172	53/171	60/164	76/148		
Female	1	0.95 (0.50, 1.78)	0.75 (0.40, 1.41)	0.42 (0.22, 0.80)^a^	0.017	
Male	1	0.88 (0.42, 1.84)	0.94 (0.45, 1.99)	0.84 (0.41, 1.70)	0.975	
Glycitein						0.011
Non-fluorosis/fluorosis	49/175	57/167	63/161	72/152		
Female	1	0.72 (0.38, 1.38)	0.82 (0.43, 1.55)	0.39 (0.20, 0.74)^a^	0.020	
Male	1	0.74 (0.35, 1.53)	0.63 (0.30, 1.31)	0.92 (0.44, 1.91)	0.987	
Genistein						0.012
Non-fluorosis/fluorosis	52/172	53/171	61/161	75/149		
Female	1	0.93 (0.49, 1.74)	0.74 (0.40, 1.39)	0.45 (0.24, 0.86)^a^	0.030	
Male	1	0.89 (0.42, 1.87)	0.88 (0.41, 1.85)	0.89 (0.44, 1.82)	0.934	
Daidzein						0.009
Non-fluorosis/fluorosis	50/174	56/168	60/164	75/149		
Female	1	0.89 (0.47, 1.69)	0.70 (0.37, 1.31)	0.41 (0.21, 0.78)^a^	0.013	
Male	1	0.74 (0.35, 1.54)	0.86 (0.40, 1.86)	0.83 (0.40, 1.69)	0.916	

OR, odds ratio; CI, confidence interval; Q, quartile. The multivariate logistic regression model was used to estimate the relationship between intake of soy isoflavones and fluorosis. Covariates adjusted for age, gender, ethnicity, marital status, education level, smoking, alcohol drinking, tea drinking, improved stove use, fuel type and using coal to roast grains and chilies, washing dry grains and chilis before use, total energy intake, calcium intake.

a *p* < 0.05.

**Table 4:** Sex-stratified analysis of the relationship between urinary levels of soy isoflavones and fluorosis OR (95% CI).

Soy isoflavones	Quartiles				*p*-trend	*p*-interaction
	Q1	Q2	Q3	Q4		
Dihydrodaidzein						0.109
Non-fluorosis/fluorosis	29/151	42/138	47/133	74/106		
Female	1	0.87 (0.40, 1.87)	0.74 (0.34, 1.59)	0.28 (0.13, 0.58)^a^	0.001	
Male	1	0.40 (0.16, 0.95)^a^	0.37 (0.16, 0.88)^a^	0.24 (0.10, 0.55)^a^	0.001	
Daidzein						0.100
Non-fluorosis/fluorosis	29/150	38/142	59/121	66/114		
Female	1	0.62 (0.27, 1.39)	0.45 (0.20, 1.00)	0.28 (0.12, 0.61)^a^	0.003	
Male	1	0.76 (0.32, 1.75)	0.32 (0.14, 0.71)^a^	0.38 (0.17, 0.84)^a^	0.004	
Glycitein						0.105
Non-fluorosis/fluorosis	35/ 144	36/144	57/123	64/116		
Female	1	0.79 (0.37, 1.70)	0.50 (0.24, 1.06)	0.44 (0.20, 0.92)^a^	0.032	
Male	1	1.40 (0.58, 3.39)	0.58 (0.27, 1.26)	0.47 (0.22, 0.99)^a^	0.014	
Equol						0.308
Non-fluorosis/fluorosis	33/146	48/132	47/133	64/116		
Female	1	0.87 (0.42, 1.82)	0.85 (0.40, 1.77)	0.55 (0.27, 1.11)	0.191	
Male	1	0.51 (0.21, 1.21)	0.44 (0.18, 1.03)	0.26 (0.11, 0.61)^a^	0.002	
Genistein						0.055
Non-fluorosis/fluorosis	40/ 139	45/135	54/126	53/127		
Female	1	0.90 (0.43, 1.92)	0.50 (0.25, 1.02)	0.59 (0.28, 1.20)	0.122	
Male	1	0.94 (0.44, 2.00)	0.90 (0.41, 1.94)	0.69 (0.32, 1.48)	0.375	
O-Desmethylangolensin				0.018		
Non-fluorosis/fluorosis	37/142	57/123	45/135	53/127		
Female	1	0.58 (0.28, 1.20)	1.25 (0.60, 2.61)	0.38 (0.18, 0.79)^a^	0.154	
Male	1	0.51 (0.23, 1.12)	0.56 (0.25, 1.28)	1.01 (0.44, 2.28)	0.771	

OR, odds ratio; CI, confidence interval; Q, quartile. The multivariate logistic regression model was used to estimate the relationship between urinary levels of soy isoflavones and fluorosis. Covariates adjusted for age, gender, ethnicity, marital status, education level, smoking, alcohol drinking, tea drinking, improved stove use, fuel type and using coal to roast grains and chilies, washing dry grains and chilis before use, total energy intake, calcium intake.

a *p* < 0.05.

### 3.6 Multivariate analysis of soy isoflavone and dental fluorosis and skeletal fluorosis

Fluorosis was divided into dental fluorosis and skeletal fluorosis, thus sensitivity analysis was conducted to assess whether the results of primary analysis were driven by type of fluorosis. Results showed associations of dietary intake and urinary levels of soy isoflavone with dental fluorosis and skeletal fluorosis were in line with fluorosis (Tables 5, 6).

**Table 5:** Odds ratios (ORs) and 95% CIs of dental fluorosis for quartiles of dietary intake and urinary levels of soy isoflavone.

Soy isoflavones	Q1	Q2	Q3	Q4	*p*
Dietary intake (mg/day)					
Non-DF/DF	56/168	72/152	80/144	89/135	
Glycitein	1	0.64 (0.41, 1.00)	0.62 (0.39, 0.96)^a^	0.52 (0.33, 0.82)^a^	0.007
Non-DF/DF	59/165	68/156	77/147	93/131	
Genistein	1	0.79 (0.51, 1.23)	0.68 (0.43, 1.06)	0.55 (0.35, 0.85)^a^	0.007
Non-DF/DF	58/166	70/154	76/148	93/131	
Daidzein	1	0.74 (0.48, 1.16)	0.68 (0.43, 1.06)	0.51 (0.33, 0.80)^a^	0.004
Non-DF/DF	59/165	68/156	76/148	94/130	
Total soy isoflavones	1	0.79 (0.50, 1.23)	0.70 (0.45, 1.10)	0.51 (0.33, 0.80)^a^	0.003
Urinary isoflavones (ng/mL)					
Non-DF/DF	46/134	47/133	69/111	76/104	
Glycitein	1	0.97 (0.58, 1.62)	0.56 (0.34, 0.92)^a^	0.46 (0.28, 0.75)^a^	<0.001
Non-DF/DF	56/124	57/123	63/117	62/118	
Genistein	1	1.01 (0.62, 1.64)	0.78 (0.48, 1.26)	0.78 (0.48, 1.26)	0.204
Non-DF/DF	39/141	54/126	67/113	78/102	
Daidzein	1	0.60 (0.36, 1.01)	0.44 (0.26, 0.74)^a^	0.34 (0.21, 0.57)^a^	<0.001
Non-DF/DF	42/138	53/127	58/122	84/96	
Dihydrodaidzein	1	0.73 (0.44, 1.21)	0.65 (0.39, 1.08)	0.34 (0.21, 0.56)^a^	<0.001
Non-DF/DF	46/134	57/123	60/120	75/105	
Equol	1	0.85 (0.51, 1.3)	0.71 (0.43, 1.17)	0.48 (0.30, 0.78)^a^	0.002
Non-DF/DF	52/128	65/115	54/126	67/113	
O-Desmethylangolensin	1	0.67 (0.42, 1.09)	1.00 (0.61, 1.63)	0.67 (0.41, 1.08)	0.300

OR, odds ratio; CI, confidence interval; DF, dental fluorosis; Q, quartile. The multivariate logistic regression model was used to estimate the association of dental fluorosis with dietary intake and urinary levels of soy isoflavone. Covariates were adjusted for age, gender, ethnicity, marital status, education level, smoking, alcohol drinking, tea drinking, improved stove use, fuel type and using coal to roast grains and chilies, washing dry grains and chilis before use, total energy intake, calcium intake.

a *p* < 0.05.

**Table 6:** Odds ratios (ORs) and 95% CIs of skeletal fluorosis for quartiles of dietary intake and urinary levels of soy isoflavone.

Soy isoflavones	Q1	Q2	Q3	Q4	*p*
Dietary intake (mg/day)					
Non-SF/SF	124/100	137/87	159/65	154/70	
Glycitein	1	0.75 (0.49, 1.14)	0.49 (0.31, 0.76)^a^	0.61 (0.39, 0.95)^a^	0.007
Non-SF/SF	121/103	143/81	156/68	154/70	
Genistein	1	0.65 (0.43, 0.99)^a^	0.51 (0.33, 0.79)^a^	0.60 (0.39, 0.93)^a^	0.012
Non-SF/SF	121/103	144/80	160/64	149/75	
Daidzein	1	0.64 (0.42, 0.97)^a^	0.47 (0.30, 0.74)^a^	0.64 (0.42, 0.99)^a^	0.020
Non-SF/SF	121/103	145/79	156/68	152/72	
Total soy isoflavones	1	0.61 (0.40, 0.94)^a^	0.51 (0.33, 0.79)^a^	0.60 (0.39, 0.93)^a^	0.015
Urinary isoflavones (ng/mL)					
Non-SF/SF	112/68	105/75	123/57	126/54	
Glycitein	1	1.13 (0.71, 1.80)	0.67 (0.41, 1.08)^a^	0.66 (0.41, 1.07)^a^	0.023
Non-SF/SF	112/68	124/56	111/69	119/61	
Genistein	1	0.86 (0.53, 1.40)	1.14 (0.72, 1.82)	0.92 (0.57, 1.48)	0.951
Non-SF/SF	108/72	105/75	117/63	136/44	
Daidzein	1	1.10 (0.69, 1.76)	0.80 (0.49, 1.29)	0.49 (0.30, 0.80)^a^	0.002
Non-SF/SF	97/83	115/65	119/61	134/46	
Dihydrodaidzein	1	0.70 (0.44, 1.12)	0.68 (0.43, 1.09)	0.42 (0.26, 0.63)^a^	0.001
Non-SF/SF	107/73	104/76	123/57	132/48	
Equol	1	1.11 (0.70, 1.77)	0.68 (0.42, 1.09)	0.56 (0.35, 0.91)^a^	0.005
Non-SF/SF	112/68	120/60	116/64	118/62	
O-Desmethylangolensn	1	0.83 (0.52, 1.34)	1.01 (0.63, 1.61)	0.88 (0.55, 1.41)	0.806

OR, odds ratio; CI, confidence interval; SF, skeletal fluorosis; Q, quartile. The multivariate logistic regression model was used to estimate the association of skeletal fluorosis with dietary intake and urinary levels of soy isoflavone. Covariates were adjusted for age, gender, ethnicity, marital status, education level, smoking, alcohol drinking, tea drinking, improved stove use, fuel type and using coal to roast grains and chilies, washing dry grains and chilis before use, total energy intake, calcium intake.

a *p* < 0.05.

## 4 Discussion

### 4.1 Summary of main findings

To the best of our knowledge, we firstly assessed the association of soy isoflavones with fluorosis. We observed that dietary intake of soy isoflavones, glycitein, daidzein and genistein were negatively associated with fluorosis. Additionally, the higher the urinary level of glycitein, daidzein, dihydrodaidzein and equol, were associated a lower occurrence of coal-burning fluorosis. Biomarkers are invaluable for providing objective, precise, and biologically relevant measures of nutrient status. Urinary isoflavone levels reflect metabolic processing and excretion, not merely dietary intake. Inter-individual metabolic differences must be considered, as they substantially influence urinary levels. However, the assessment of dietary soy isoflavones through FFQ can reflect the dietary levels of long-term habits, which is very important for exploring the relationship between dietary isoflavone and disease. In the present study, we investigated both dietary and urinary exposure of soy isoflavones, and we found both internal and external exposure levels of soy isoflavones was negatively associated the occurrence of fluorosis. These rigorous findings suggested that higher exposure level of soy isoflavones is associated with the lower the risk of coal-burning fluorosis. An experimental animal study on the effects of multi-nutrient intervention in coal-burning fluorosis on dental fluorosis, bone fluoride and urinary fluoride in female rats suggested that multi-element plus soy intervention resulted in lower bone fluoride levels (23). This may be attributed to the fact that soy foods are rich in soy isoflavones. A five-year cohort study (*n* = 1,587) found a 1.95% reduction in bone mineral density (BMD) and a 69% increased risk of osteoporosis (95% CI: 1.09, 2.61) in the lowest group compared to the highest quartile of soy isoflavones (≥62.64 mg/day) (24). In addition, studies have shown that increasing the intake of soy products, soy protein or soy isoflavones can improve bone resorption or reduce bone loss (14, 25). A recent meta-analysis of 26 randomized controlled trials (*n* = 2,652) also found that supplementation of soy isoflavones had a strong beneficial effect on estrogen deficient bone loss in women (26), and intervention of soy isoflavones can effectively improve lumbar spine and total femur bone mineral density in women (27, 28). Therefore, both observational and interventional studies have shown that soy isoflavones have beneficial effects on bone health. In this study, dietary and urinary soy isoflavones were found to be negatively correlated with fluorosis, which proves that the beneficial effects of soy isoflavones on bone health may antagonize the effects of fluorosis on bone injury. It is acknowledged that urinary isoflavone levels reflect metabolic processes and excretion rather than intake alone, and inter-individual metabolic variations affect these levels, which will be addressed in future studies.

### 4.2 Explanation of biological mechanisms

Excessive fluoride induces oxidative stress in bone tissues by increasing the content of reactive oxygen species (ROS) and lipid peroxides (MDA) in bone tissues and reducing the activity of antioxidant enzymes (20, 29). Although oxidative stress indicators were not directly measured, the antioxidant properties of isoflavones allow us to infer that they alleviate fluorosis by reducing oxidative stress damage. Soy isoflavones can affect fluorosis through their antioxidant properties because they are well-documented antioxidant substances. In recent years, studies have shown that soy isoflavones significantly elevated total antioxidant capacity and superoxide dismutase enzyme activities, decreased malondialdehyde level, promoting antioxidant element nuclear erythroid-2-related factor 2, and its downstream targets, including heme oxygenase 1, and quinone oxidoreductase 1 protein expressions (30). Furthermore, isoflavones can enhance bone formation and inhibit bone resorption by influencing the cell signaling pathways of osteoblast and osteoclast differentiation, including the Wnt-β-catenin and BMP pathways that stimulate bone formation (31). In addition, studies have shown that soy isoflavones are phytoestrogens with physiological functions such as preventing osteoporosis, increasing bone density and bone strength in rats with osteoporotic fractures, promoting the expression of vascular endothelial growth factor and osteoprotegerin at the fracture end, improving the histological environment, and promoting the healing of osteoporotic fractures (32, 33). Notably, although we detected dietary and urinary soy isoflavones and used multivariate analyses to demonstrate negative association, we did not measure urinary bone turnover markers, limiting direct evidence for bone health conclusions. Overall, the negative associations between soy isoflavones and fluorosis may be justified based on the fact that they act as antioxidants to reduce oxidative stress damage and have a positive effect on fluorosis. However, further studies are needed to elucidate the pathophysiological mechanism.

### 4.3 Gender-specific associations and the role of estrogen

In this study, stratified analysis showed soy isoflavones may be inversely associated with fluorosis in women, but not in men, indicating associations between soy isoflavones and fluorosis may be modified by gender. Some related studies have shown that the reduction of BMD loss by soy protein is mainly due to the phytoestrogenic effects of soy isoflavones (34, 35). A clinical study has shown that soy isoflavones can improve bone mineral density and prevent bone calcium loss in postmenopausal women, and soy isoflavones can also significantly slow down the bone turnover rate in postmenopausal women, which is beneficial to maintaining the dynamic balance of bone resorption-bone formation, and has the effect of preventing and treating osteoporosis in postmenopausal women (36). Sex-specific associations were also observed in other studies, such as colorectal cancer (37) and type 2 diabetes (38). The bases for the sex-specific effects may be soy isoflavones have at least two hydroxyl functions in opposite positions on the molecule, which mimics effect of estradiol. This may be attributed to the interaction between estrogen receptor expression levels in women and the phytoestrogenic effects of the isoflavones. Isoflavones not only act as weak estrogens but also compete with endogenous estrogen for binding to the estrogen receptor (39). ERα activates intracellular phosphorylation pathways including PI3K/Akt and NF-κB and exerting its biological effect (40). Additionally, menopausal status as a potentially important confounder in interpreting sex-specific findings. Postmenopausal women experience estrogen decline, whereas studies have demonstrated that soy isoflavone supplementation improves menopausal symptoms and postmenopausal bone health (41–43). Thus, postmenopausal estrogen decline can be partially offset by soy isoflavones acting as phytoestrogens. However, it was regrettable that we did not collect this status due to the estrogen levels of women gradually decrease after menopause. Future epidemiological studies need to determine whether estrogen biomarkers or menopausal status affect the effects of soy isoflavones on fluorosis risk.

### 4.4 Study limitations and directions for future research

Although this study has built upon existing literature, it is not without limitations. First, the cross-sectional design inherently restricts our ability to establish a causal relationship between isoflavones and fluorosis. However, we implemented stringent inclusion and exclusion criteria during participant recruitment to mitigate potential reverse causality to some extent. Second, while the Food Frequency Questionnaire (FFQ) may introduce measurement errors, the questionnaire utilized in this study has been validated, and we conducted a multifactorial analysis to minimize the influence of these errors on our findings. Third, although patients diagnosed with fluorosis may alter their dietary habits, such changes do not result in misclassification of their fluorosis status, allowing for some degree of generalizability of our results. Fourth, gender-stratified analysis was conducted, female menopausal status was not stratified due to we did not collect menopausal status during the investigation. Potential hormonal influences on the observed relationships was not explored. Future studies should collect data on menopausal status to clarify hormone-related effects. Additionally, we did not measure urinary bone turnover markers, limiting direct evidence for bone health conclusions. Finally, although we adjusted for major sociodemographic characteristics, lifestyle factors, dietary factors, confounding by unknown or unmeasured factors cannot be completely ruled out, such as hormonal level, bone turnover markers. Future research should focus on these issues. (1) Individual metabolic variations affect urinary soy isoflavone levels, how do metabolism-related gene polymorphisms affect the association between soy isoflavones and fluorosis? (2) How do menopausal status or hormone levels affect the association between soy isoflavones and fluorosis? (3) How do bone turnover markers affect the association between soy isoflavones and fluorosis? Therefore, In the future, large-scale prospective studies should be carried out to collect menopausal status, detect levels of hormone and bone turnover marker, and determine gene of the metabolic pathway and bone turnover, in order to clarify their roles in the relationship between soy isoflavones and fluorosis. Moreover, further studies are needed to elucidate the pathophysiological mechanism by animal intervention experiments.

## 5 Conclusion

The higher exposure level of soy isoflavones is associated with the lower the risk of coal-burning fluorosis. The study provides evidence-based support for developing dietary recommendations incorporating soy isoflavones to help reduce fluorosis risk in endemic areas. Thus, we advocate increasing dietary soy isoflavone intake, particularly in coal-burning fluorosis areas. Food manufacturers should develop high-isoflavone fortified foods for high-risk population. Multi-sectoral collaboration is needed to implement prevention strategies at the grassroots level, complemented by health education to enhance public self-protection awareness.

## Data Availability

The raw data supporting the conclusions of this article will be made available by the authors, without undue reservation.

## References

[1] Zeng A Li H Hu X Yang M Mu M Chen Y. Fibroma development in rats with dental fluorosis: activation pathways of endoplasmic reticulum stress and mitochondria. J Zunyi Med Univ. (2021) 44:433–9. doi: 10.14169/j.cnki.zunyixuebao.2021.0071

[2] Zhou J Sun D Wei W. Necessity to pay attention to the effects of low fluoride on human health: an overview of skeletal and non-skeletal damages in epidemiologic investigations and laboratory studies. Biol Trace Elem Res. (2023) 201:1627–38. doi: 10.1007/s12011-022-03302-7, PMID: 35661326

[3] Guan Z Wu C Qi X. Evaluation of the health status of the population in coal fired fluorosis areas in Guizhou Province after comprehensive management. J Guizhou Med Univ. (2018) 43:1124–8. doi: 10.19367/j.cnki.1000-2707.2018.10.001

[4] Fan S. The current situation of endemic fluorosis prevention and control in China during the 13th five-year plan period. OEM. (2020) 37:1219–23. doi: 10.13213/j.cnki.jeom.2020.20274

[5] Wang X Gao J Li C Song L Zhang X Pan X. Changes of fluorine exposure level of population in endemic fluorosis areas caused by coal burning in Guizhou Province. E&H. (2020) 37:620–2. doi: 10.16241/j.cnki.1001-5914.2020.07.012

[6] Wang M Shen H Chen X Li Y Zhou X La C. Meta-analysis on the effectiveness of health education interventions for endemic fluorosis in China. CJCED: Chin J Control Endem Dis. (2024) 39:16–9.

[7] Jianyang G Hongchen W Zhiqi Z Jingfu W Haiqing L. Review on health impacts from domestic coal burning: emphasis on endemic fluorosis in Guizhou province, Southwest China. RECT. (2021):258. doi: 10.1007/398_2021_71 34625836

[8] Huo X Wang S Chen Z. Effect of soy isoflavones on oxidative hydrogen peroxide-induced stress in IPEC-J2 cells. CJAVS: Guangdong J Anim Vet Sci. (2021) 46:37–40.

[9] Yari Z Tabibi H Najafi I Hedayati M Movahedian M. Effects of soy isoflavones on serum systemic and vascular inflammation markers and oxidative stress in peritoneal dialysis patients: a randomized controlled trial. Phytother Res. (2020) 34:3011–8. doi: 10.1002/ptr.6729, PMID: 32419281

[10] Xiong X Xu Z He N He J Chen Z Huang C. Intervention of soy isoflavones in hepatic oxidative stress and inflammation in obese rats. Acta Agric Zhejiangensis. (2022) 34:942–8.

[11] Greendale GA Tseng CH Han W Huang M-H Leung K Crawford S. Dietary isoflavones and bone mineral density during midlife and the menopausal transition: cross-sectional and longitudinal results from the study of women’s health across the nation phytoestrogen study. Menopause. (2015) 22:279–88. doi: 10.1097/GME.0000000000000305, PMID: 25116050 PMC4324399

[12] Barańska A Kanadys W Bogdan M Stępień E Barczyński B Kłak A. The role of soy isoflavones in the prevention of bone loss in postmenopausal women: a systematic review with meta-analysis of randomized controlled trials. J Clin Med. (2022) 11:4676. doi: 10.3390/jcm11164676, PMID: 36012916 PMC9409780

[13] Kanadys W Barańska A Błaszczuk A Polz-Dacewicz M Drop B Malm M. Effects of soy isoflavones on biochemical markers of bone metabolism in postmenopausal women: a systematic review and meta-analysis of randomized controlled trials. Int J Environ Res Public Health. (2021) 18:5346. doi: 10.3390/ijerph18105346, PMID: 34067865 PMC8156509

[14] Akhlaghi M Ghasemi Nasab M Riasatian M Sadeghi F. Soy isoflavones prevent bone resorption and loss, a systematic review and meta-analysis of randomized controlled trials. Crit Rev Food Sci. (2020) 60:2327–41. doi: 10.1080/10408398.2019.1635078 31290343

[15] El-Demerdash A Atanasov A Bishayee A Abdel-Mogib M Hooper JNA Al-Mourabit A. *Batzella*, *Crambe* and *Monanchora*: highly prolific marine sponge genera yielding compounds with potential applications for cancer and other therapeutic areas. Nutrients. (2018) 10:33. doi: 10.3390/nu10010033 29301302 PMC5793261

[16] Al-Bari MAA Hossain S Mia U Al Mamun MA. Therapeutic and mechanistic approaches of *Tridax procumbens* flavonoids for the treatment of osteoporosis. Curr Drug Targets. (2020) 21:1687–702. doi: 10.2174/1389450121666200719012116, PMID: 32682372

[17] Qiao L Liu X He Y Zhang J Huang H Bian W. Progress of signaling pathways, stress pathways and epigenetics in the pathogenesis of skeletal fluorosis. Int J Mol Sci. (2021) 22:1932. doi: 10.3390/ijms222111932, PMID: 34769367 PMC8584317

[18] Zhu J Liu J Zhao W Zhang H. Protective effect of soy isoflavones on liver and kidney function injury in rats with fluorosis. CJCED: Chin J Control Endem Dis. (2016) 31:527.

[19] Qin X Wang S Yu M Zhang L Li X Zuo Z. Child skeletal fluorosis from indoor burning of coal in southwestern China. J Environ Public Health. (2009) 2009:969764. doi: 10.1155/2009/969764, PMID: 20041010 PMC2778178

[20] Liu J Yang S Luo MJ Chen T Ma XJ Tao N. Association between dietary patterns and fluorosis in Guizhou, China. Front Nutr. (2019) 6:189. doi: 10.3389/fnut.2019.00189, PMID: 32039225 PMC6985547

[21] Cui Q Xia Y Wu Q Chang Q Niu K Zhao Y. Validity of the food frequency questionnaire for adults in nutritional epidemiological studies: a systematic review and meta-analysis. Crit Rev Food Sci. (2023) 63:1670–88. doi: 10.1080/10408398.2021.1966737, PMID: 34520300

[22] Yang Y. Chinese food ingredient list. Beijing: Peking University Press (2022).

[23] Yu S Wang F Xia S. Effects of multi-nutrient intervention on dental fluorosis, bone fluoride and urinary fluoride in female rats with coal burning fluorosis. E&H. (2017) 34:477–80.

[24] Baglia ML Gu K Zhang X Zheng Y Peng P Cai H. Soy isoflavone intake and bone mineral density in breast cancer survivors. Cancer Cause Control. (2015) 26:571–80. doi: 10.1007/s10552-015-0534-3 PMC4368486 25687481

[25] Tang S Du Y Oh C No J. Effects of soy foods in postmenopausal women: a focus on osteosarcopenia and obesity. J Obes Metab Syndr. (2020) 29:180–7. doi: 10.7570/jomes20006, PMID: 32843586 PMC7539339

[26] MNT L Hu LM Jeppesen PB. A systematic review and meta-analysis of the effects of isoflavone formulations against estrogen-deficient bone resorption in peri- and postmenopausal women. Am J Clin Nutr. (2017) 106:801–11. doi: 10.3945/ajcn.116.151464 28768649

[27] Shams-White MM Chung M Du M Fu Z Insogna KL Karlsen MC. Dietary protein and bone health: a systematic review and meta-analysis from the National Osteoporosis Foundation. Am J Clin Nutr. (2017) 105:1528–43. doi: 10.3945/ajcn.116.145110 28404575

[28] Zheng X Lee SK Chun OK. Soy isoflavones and osteoporotic bone loss: a review with an emphasis on modulation of bone remodeling. J Med Food. (2016) 19:1–14. doi: 10.1089/jmf.2015.0045, PMID: 26670451 PMC4717511

[29] Babu S Manoharan S Ottappilakkil H Perumal E. Role of oxidative stress-mediated cell death and signaling pathways in experimental fluorosis. Chem Biol Interact. (2022) 365:110106. doi: 10.1016/j.cbi.2022.110106, PMID: 35985521

[30] Lu C Wei Z Jiang N Chen Y Wang Y Li S. Soy isoflavones protects against cognitive deficits induced by chronic sleep deprivation via alleviating oxidative stress and suppressing neuroinflammation. Phytother Res. (2022) 36:2072–80. doi: 10.1002/ptr.7354, PMID: 35373399

[31] Nakai S Fujita M Kamei Y. Health promotion effects of soy isoflavones. J Nutr Sci Vitaminol. (2020) 66:502–7. doi: 10.3177/jnsv.66.502, PMID: 33390391

[32] Gómez-Zorita S González-Arceo M Fernández-Quintela A Eseberri I Trepiana J Portillo MP. Scientific evidence supporting the beneficial effects of isoflavones on human health. Nutrients. (2020) 12:3853. doi: 10.3390/nu12123853, PMID: 33348600 PMC7766685

[33] Ao R Zhang J Gu J Yu T Liang W Zhang T. The effect of soy isoflavones on osteoporotic fracture healing in ovariectomized rats. J Orthop. (2015) 18:110–3.

[34] Sathyapalan T Aye M Rigby AS Fraser WD Thatcher NJ Kilpatrick ES. Soy reduces bone turnover markers in women during early menopause: a randomized controlled trial. J Bone Miner Res. (2017) 32:157–64. doi: 10.1002/jbmr.2927, PMID: 27465911

[35] Yelland S Steenson S Creedon A Stanner S. The role of diet in managing menopausal symptoms: a narrative review. Nutr Bull. (2023) 48:43–65. doi: 10.1111/nbu.12607, PMID: 36792552

[36] Inpan R Dukaew N Na Takuathung M Teekachunhatean S Koonrungsesomboon N. Effects of isoflavone interventions on bone turnover markers and factors regulating bone metabolism in postmenopausal women: a systematic review and meta-analysis of randomized controlled trials. Arch Osteoporos. (2024) 20:2. doi: 10.1007/s11657-024-01467-3 39708251

[37] Khankari NK Yang JJ Sawada N Wen W Yamaji T Gao J. Soy intake and colorectal cancer risk: results from a pooled analysis of prospective cohort studies conducted in China and Japan. J Nutr. (2020) 150:2442–50. doi: 10.1093/jn/nxaa194, PMID: 32692347 PMC7762761

[38] Woo HW Kim MK Lee YH Shin DH Shin MH Choi BY. Sex-specific associations of habitual intake of soy protein and isoflavones with risk of type 2 diabetes. Clin Nutr. (2021) 40:127–36. doi: 10.1016/j.clnu.2020.04.035, PMID: 32418714

[39] Wang Y Tian M He H Du J. Meta-analysis of safety of soy isoflavones in postmenopausal women. J Toxicol. (2018) 32:10. doi: 10.16421/j.cnki.1002-3127.2018.04.005

[40] Canivenc-Lavier MC Bennetau-Pelissero C. Phytoestrogens and health effects. Nutrients. (2023) 15:317. doi: 10.3390/nu15020317, PMID: 36678189 PMC9864699

[41] Pokushalov E Ponomarenko A Garcia C Kasimova L Pak I Shrainer E. Assessing the combined effects of black cohosh, soy isoflavones, and SDG lignans on menopausal symptoms: a randomized, double-blind, placebo-controlled clinical trial. Eur J Nutr. (2025) 64:138. doi: 10.1007/s00394-025-03588-y, PMID: 40131516

[42] Zhou Y Su Z Liu G Hu S Chang J. The potential mechanism of soy isoflavones in treating osteoporosis: focusing on bone metabolism and oxidative stress. Phytother Res. (2025) 39:1645–58. doi: 10.1002/ptr.8451, PMID: 39921597

[43] Cui Y Cai H Gao Y Dai Q Yang G Zheng W. Associations of dietary intakes of calcium, magnesium and soy isoflavones with osteoporotic fracture risk in postmenopausal women: a prospective study. J Nutr Sci. (2022) 11:e62. doi: 10.1017/jns.2022.52, PMID: 35992572 PMC9379929

