# Supplementary material for: A cross-sectional study of dietary and urinary soy isoflavones about coal-burning fluorosis in Guizhou, China
Source: Front Nutr. 2025 Jul 24;12:1589177. doi: 10.3389/fnut.2025.1589177 (PMC12328192; doi:10.3389/fnut.2025.1589177)

1. Supplementary Figure Legend

Supplementary Figure 1: Sex-stratified analysis of the relationship between soy isoflavones and fluorosis and interactive effects. A: Sex-stratified analysis of the relationship between dietary intake of soy isoflavones and fluorosis and interactive effects. B: Sex-stratified analysis of the relationship between urinary level of soy isoflavones and fluorosis and interactive effects. Abbreviations: *CI*, confidence interval. Q, quartile. *P*-interaction: Values indicate the statistical significance of interaction effects between sex and soy isoflavone intake on fluorosis risk. Covariates variable adjusted for age, sex, marital status, education level, income, smoking status, alcohol drinking status, tea drinking status, using coal to roast grains or chili, washing dry grains or chili before use, fuel type, using improved stove, calcium intake, roasted chili and grains consumption, and total energy intake.

Supplementary Figure 1: Sex-stratified analysis of the relationship between urinary levels of soy isoflavones and fluorosis and interactive effects.

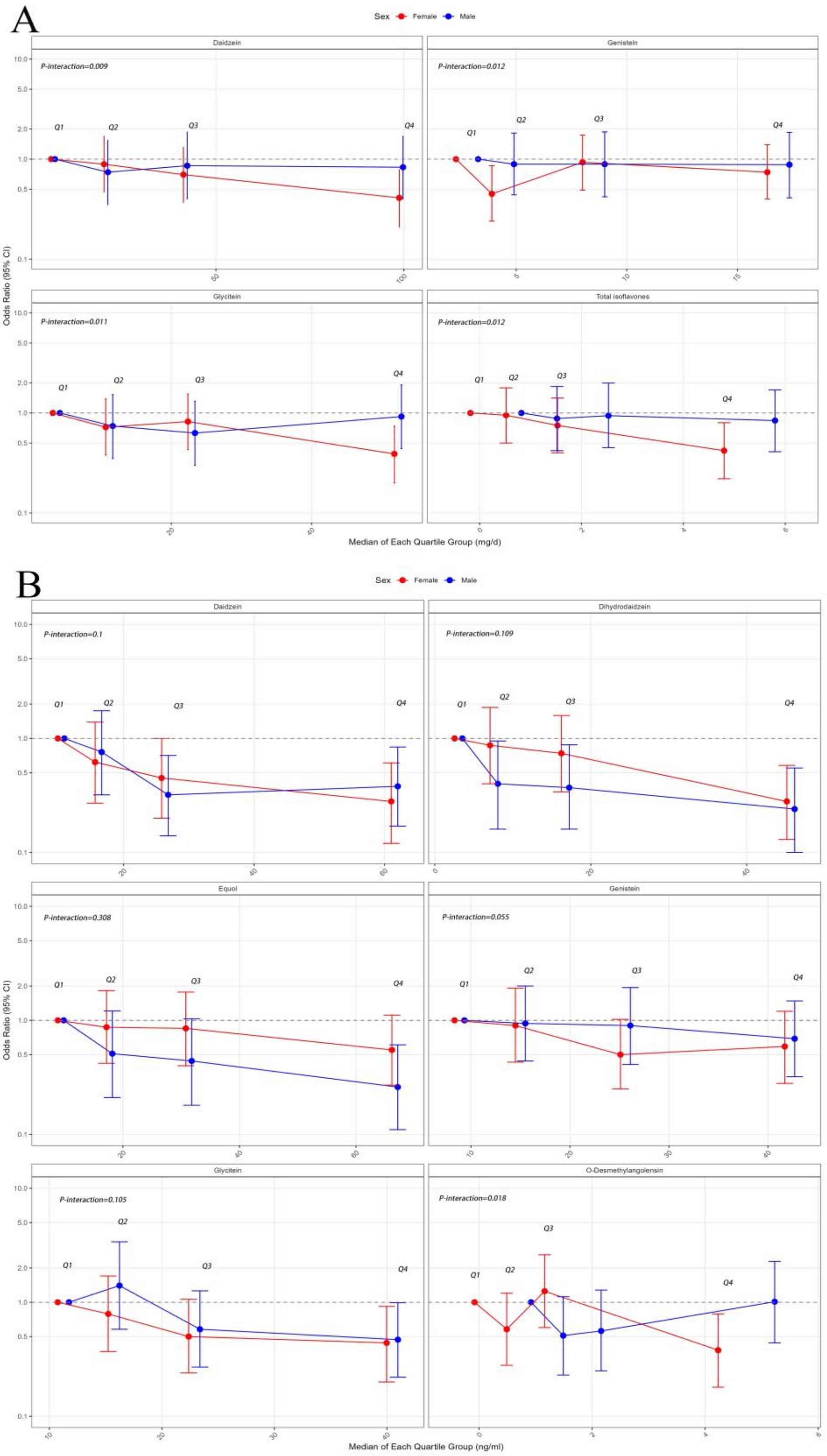

Supplement: Supplementary file 1 [file Data_Sheet_1.pdf]
